# Adenomatoid odontogenic tumour associated with dentigerous cyst - unusual case report

**DOI:** 10.1016/S1808-8694(15)31135-6

**Published:** 2015-10-20

**Authors:** Cassiano Francisco Weege Nonaka, Lélia Batista de Souza, Lêda Bezerra Quinderé

**Affiliations:** 1 MS. Dentist; 2 PhD in Oral Pathology - Programa de Pós-Graduação em Patologia Oral da Universidade Federal do Rio Grande do Norte-UFRN; 3 PhD in Oral Pathology; Professor of pediatric dentistry - Universidade Federal do Rio Grande do Norte

**Keywords:** dentigerous cyst, odontogenic tumour, adenomatoid odontogenic tumour

## Abstract

The adenomatoid odontogenic tumor is a relatively uncommon lesion which mainly affects females in their second decade of life, exhibiting predilection for the anterior region of the maxilla. The lesion is usually associated with the crown of an enclosed tooth, most commonly the maxillary canine. In this paper we present a case of adenomatoid odontogenic tumor associated with a dentigerous cyst affecting the left maxillary region in a 13-year-old female. The authors also discuss clinical, radiographic, histopathologic and therapeutic features of the case.

## INTRODUCTION

The adenomatoid odontogenic tumor is a benign tumor that is keen to involve the anterior region of the maxillary bones, with a larger number of cases in females, in their second decade of life, and that may, sporadically, be associated with odontogenic cystic lesions. The present study aims at reporting an uncommon case of adenomatoid odontogenic tumor, associated with a dentigerous cyst, as well as discussing the clinical, radiographic, histopathological and therapeutic characteristics related to the case.

## CASE REPORT

A 13 year old female had a rigid mass in the oral cortical bone of her maxilla, with mucosa integrity, and lacking teeth 23 and 24 on the affected side. Panorama radiographic study revealed a radiolucent lesion, circumscribed, with few radiopaque areas inside, associated to the canine tooth, which was shifted towards the nasal cavity.

**Figure 1:**
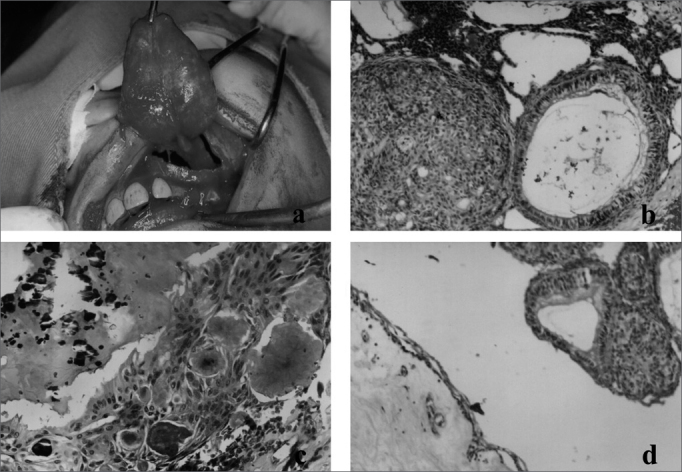
(a) Lesion enucleation including the unerupted canine; (b) Adenomatoid odontogenic tumor histological aspect, stressing the proliferation of fusiform/globular cells arranged as large islands and ductiform structures (H/E 100x); (c) Microscopic aspect showing foci of eosinophilic amorphous material and calcification areas (H/E 200x); (d) Photomicrography showing a cystic lesion coated by stratified pavement epithelium, formed by few cell layers, associated to adenomatoid odontogenic tumor (H/E 100x).

We carried out an intraoral surgical intervention through a Neumann's incision, from the alveolar and left central superior incisive gingiva all the way to the first ipsilateral molar, shifting the mucoperiosteum. After that, the tumor mass and the unerupted canine were removed (Figure 1a), and finally we repositioned the tissue and sutured the mucoperiosteal flap.

The material removed was then sent to the Pathology Department of the Oral Pathology Division of the Dentistry School of UFRN. After routine histology on the material, under light microscopy they observed fragments of the odontogenic lesion, characterized by the proliferation of fusiform/globular cells, arranged as large islands and solid sheets, as well as numerous structures similar to ducts, lined by low cylindrical or cubic cells, of polarized nuclei (Figure 1b). Occasionally, some detached eosinophylic amorphous material was, together with calcification areas (Figure 1c). We also noticed the presence of a cystic lesion coated by a stratified pavement epithelium, formed by few cell layers (Figure 1d), showing a continuity with the neoplastic foci aforementioned, and a connective fibrous capsule. Histological diagnosis was of Adenomatoid Odontogenic tumor, associated with a dentigerous cyst.

After 12 months, the patient returned to our department, not showing clinical or radiographic signs of tumor recurrence. We did notice bone remodeling and neoformation.

## DISCUSSION

The clinical findings presented in this case corroborate characteristic reports present in the literature, such as a higher incidence in women1, especially within the second decade of life 2, and the maxilla lesion associated with an erupted tooth^1^,^2^.

The radiographic findings presented hereby are peculiarities of the adenomatoid odontogenic tumor follicular variant, presenting itself as a radiolucent unilocular lesion, well outlined, associated to the crown of the unerupted tooth^3^,^4^. Mild radiopaque foci were seen inside the lesion by means of the panoramic radiography, such characteristic is seen in over 50% of the cases of adenomatoid odontogenic tumors^1^,^5^.

Few cases of adenomatoid odontogenic tumors associated with dentigerous cysts are reported in the literature. Santos et al.^5^ reported a case of adenomatoid odontogenic tumor being developed in the fibrous capsule of the dentigerous cyst. Garcia-Pola et al.^4^ described the prolipheration of an adenomatoid odontogenic cyst in the epithelial border of a dentigerous cyst. Prolipheration histopathological findings from the cystic epithelial border and the fibrous capsule, similar to what was described by the aforementioned authors was also observed in the case described by the present paper.

Because of its low tendency to recur, the case hereby presented was treated by conservative surgical enucleation associated with the removal of the unerupted dental element, and such procedure was proposed in numerous papers4,6. In favorable cases, dentigerous cyst associated adenomatoid odontogenic tumor marsupialization provide a conservative character to the treatment, and allow the later eruption of the dental element^6^.

## FINAL COMMENTS

There are very few cases of adenomatoid odontogenic tumors associated with dentigerous cysts reported in the literature. The present investigation describes a case of adenomatoid odontogenic tumor associated with a dentigerous cyst, highlighting the importance of the histopathological exam in the cystic lesions of the maxillary bones. Moreover, we stress the conservative surgical intervention as a treatment of option for adenomatoid odontogenic tumors.
